# Adipose-Derived Stem Cell Therapy in Spinal Cord Injury

**DOI:** 10.3390/cells13171505

**Published:** 2024-09-09

**Authors:** Jad El Masri, Hiba Fadlallah, Rahaf Al Sabsabi, Ahmad Afyouni, Mohamed Al-Sayegh, Wassim Abou-Kheir

**Affiliations:** 1 Department of Anatomy, Cell Biology, and Physiological Sciences, American University of Beirut, Beirut 1107-2020, Lebanon; jse20@mail.aub.edu (J.E.M.); hjf05@mail.aub.edu (H.F.); 2 Faculty of Medical Sciences, Lebanese University, Beirut 1533, Lebanon; alsabsabirahaf@gmail.com (R.A.S.); ahmadafyouni2000@gmail.com (A.A.); 3 Biology Division, New York University Abu Dhabi, Abu Dhabi 2460, United Arab Emirates

**Keywords:** adipose tissue, spinal cord injury, stem cells, transplantation

## Abstract

Spinal cord injury (SCI) is a serious condition accompanied by severe adverse events that affect several aspects of the patient’s life, such as motor, sensory, and functional impairment. Despite its severe consequences, definitive treatment for these injuries is still missing. Therefore, researchers have focused on developing treatment strategies aimed at ensuring full recovery post-SCI. Accordingly, attention has been drawn toward cellular therapy using mesenchymal stem cells. Considering their wide availability, decreased immunogenicity, wide expansion capacity, and impressive effectiveness in many therapeutic approaches, adipose-derived stem cell (ADSC) injections in SCI cases have been investigated and showed promising results. In this review, SCI pathophysiology and ADSC transplantation benefits are discussed independently, together with SCI animal models and adipose stem cell preparation and application techniques. The mechanisms of healing in an SCI post-ADSC injection, the outcomes of this therapeutic approach, and current clinical trials are also deliberated, in addition to the challenges and future perspectives, aiming to encourage further research in this field.

## 1. Introduction

Spinal cord injury (SCI) is an unfortunate incident that can affect any part of the spinal cord (cervical, thoracic, lumbar, and sacral). Since they are typically accompanied by several motor, sensory, functional, and psychological adverse events, SCIs may significantly affect the patient’s quality of life. Every year, approximately 250,000–500,000 individuals suffer from an SCI, with varying prevalence in different countries [1,2]. SCIs also have a male predominance with a male–female ratio of 4:1 in the United States [3].

The underlying cause behind SCIs may sometimes be non-traumatic, such as spinal metastases, inflammatory illnesses, and degenerative disorders [4]. In other cases, SCIs are induced by traumatic events at the level of the spinal cord, most frequently from falls and motor vehicle accidents [5]. This explains why people above 65 years of age and those in their late teens or early adulthood are most commonly affected. However, SCIs can occur at any age [6].

SCI can be classified as either complete or incomplete based on the depth and extent of the injury. A complete SCI is when the entire area below the injury site is affected and no electrical impulses are traveling beyond it, resulting in a complete loss of motor, sensory, and functional abilities in this area. Conversely, in an incomplete SCI, some of these abilities are preserved, which makes it less severe [6,7].

Since SCIs are often the result of accidents, prophylactic treatment is not possible, and prevention is limited to precautionary measures, such as wearing helmets [6,7]. For this reason, researchers are focusing on methods of achieving any possible recovery following SCIs [8]. Several therapeutic approaches have been used such as those targeting blood pressure control, in addition to high-dose steroid use. However, these interventions have failed to fully treat SCI patients [9].

The employment of cutting-edge treatments by research teams has resulted in significant advances in neuroscience and regenerative medicine, which has created new opportunities aiming at complete SCI recovery using cellular therapy. Given that the mesenchymal stem cell (MSC) injection targets many of the areas involved in the injury’s pathophysiology [10], experimental and clinical trials have been invested in studying the benefits of different MSC transplantations (bone marrow, umbilical cord, and adipose-derived MSCs) in the setting of an SCI [11]. For example, MSC grafts can help establish a microenvironment that promotes tissue repair and regeneration by regulating the neuroinflammation mediated by macrophages, astrocytes, and T lymphocytes. Thus, they may have promising benefits by modulating inflammation, one of the major problems encountered in the setting of SCIs [12].

In light of this, fatty tissue is one of the main sources of these stem cells used in research, since adipose-derived stem cells (ADSCs) have less immunogenicity and an outstanding capacity for expansion, in addition to their wide availability and their cellular collection by the means of less invasive methods [13,14]. ADSCs have been proven to be both safe and rewarding when transplanted for several purposes, including aesthetic reconstructive surgeries [15], chronic ulcer treatment in patients with lower-extremity arteriopathies, and neural regeneration, such as in the case of an SCI [16,17].

In light of this, in our review, we aimed to delve deeply into the complexities of SCI and adipose stem cell transplantation. Therefore, our information-gathering process involved a thorough review of the published literature. First, we explained the different pathophysiological mechanisms underlying SCIs. We also explored the mechanism of action of ADSCs. We also collected the available data on animal models used in SCI experiments, in addition to the ADSC application methods. Lastly, the mechanism of healing in SCI cases post-ADSC graft and its functional, motor, and sensory outcomes were extensively explored.

## 2. Pathophysiology of Spinal Cord Injury

Understanding the pathophysiology of SCIs is crucial for comprehending the role of ADSC transplantation in promoting functional recovery. There are two major components of the pathophysiology.

### 2.1. Primary Injury

#### 2.1.1. Non-Traumatic SCI

The pathophysiology of non-traumatic spinal cord injury (NTSCI) is highly variable and depends largely on the underlying etiology [18]. However, common pathogenic secondary injury mechanisms mirror those observed in traumatic spinal cord injuries but without the high-energy primary injury [19]. In addition, degenerative spinal column conditions are among the most prevalent causes of acquired non-traumatic spinal cord injury, leading to progressive spinal cord compression and subsequent neurological deficits [20]. Moreover, various comorbidities (i.e., diabetes, cardiovascular diseases, and cognitive impairment) seen in older patients with NTSCI, further complicate pathophysiological processes [21].

#### 2.1.2. Traumatic SCI

Acute spinal cord injury (SCI) arises from sudden trauma to the spine, resulting in burst fractures, dislocation of vertebrae, and damage to various spinal structures. The primary injury phase, marked by the tearing of bone fragments and spinal ligaments, involves damage to the cellular membrane, the destruction of neural tissue, disruption of the axonal network, hemorrhage, and damage to the glial membrane [22,23,24,25].

### 2.2. Secondary Injury

The primary injury initiates a secondary injury that inflicts additional chemical and mechanical damage on spinal tissues, resulting in neuronal excitotoxicity due to elevated calcium accumulation, increased reactive oxygen concentrations, and heightened glutamate levels. These events cause damage to underlying nucleic acids, proteins, and phospholipids, ultimately resulting in neurological dysfunction [26]. The secondary injury phase unfolds in three distinct phases: acute, sub-acute, and chronic. In the acute phase, clinical manifestations of secondary injury include increased cell permeability, ischemia, vascular damage, edema, excitotoxicity, ionic deregulation, inflammation, and free radical formation [22,27]. If the acute secondary injury persists, the sub-acute phase ensues, which is characterized by features such as neuronal apoptosis, axonal demyelination, Wallerian degeneration, and fibroglial scar formation [22,27]. Should the sub-acute secondary injury continue, the chronic phase initiates, leading to cyst formation, axonal dieback, and maturation of the glial scar [22,28]. In the following section, the pathophysiology of each phase is thoroughly discussed.

After the primary injury, the most critical clinical manifestation is the interruption of spinal cord vascular supply, leading to ischemia [27,28]. This pathophysiological state provokes a disturbance in the water-solute balance in the intracellular compartment, which subsequently leads to cellular edema and promotes cell death [22]. Moreover, rupture of capillaries due to the primary injury promotes the extravasation of leukocytes and red blood cells [22]. Inflammatory cytokines, such as TNF, IL-1, and IL-6, are extensively released 6 to 12 h post-injury by the microglia, peripherally derived macrophages, and neutrophils, leading to additional cell death [22,29]. The presence of glial fibrillary acidic proteins (GFAP) and IL-6 in the cerebrospinal fluid (CSF) indicates the presence of neuroinflammation leading to cell necrosis [30].

Glutamate-mediated excitotoxicity and neurotoxicity is provoked by the hyper-activation of NMDA and AMPA receptors, which further promotes apoptosis and necrosis [31]. Glutamate increases intracellular Na^+^ and Ca^2+^ concentrations, thereby inhibiting mitochondrial respiration, resulting in reactive oxygen species (ROS) generation and disturbance of ionic homeostasis, causing further cell cytotoxic edema [22,31].

High Ca^2+^ levels in the neurons increase the risk of acute axonal degeneration [32]. The Wallerian degeneration is an anterograde phenomenon that occurs 24 to 48 h after the injury and is manifested by the formation of retraction bulbs that inhibit axonal regeneration [33]. The retrograde degeneration of axons is termed “axonal dieback” and occurs in the chronic phase [30].

Glial scar formation, or gliosis, is a reactive cellular mechanism facilitated by astrocytes during the chronic secondary phase of SCI. Astrogliosis, the scarring of astrocytes, represents the body’s natural process aimed at shielding and initiating the healing process following SCI [34]. Pericytes are the other major constituent of scar tissue; they promote fibroblast extracellular matrix expression (i.e., fibronectin) [35].

The continual enlargement of the SCI site results in the formation of a cyst, signaling ongoing apoptotic responses. During this process, astrocytes undergo necroptosis cell death through TLR4/MyD88 signaling [36]. Syringomyelia can be seen, inducing further damage to the spinal cord [22].

**Figure 1:**
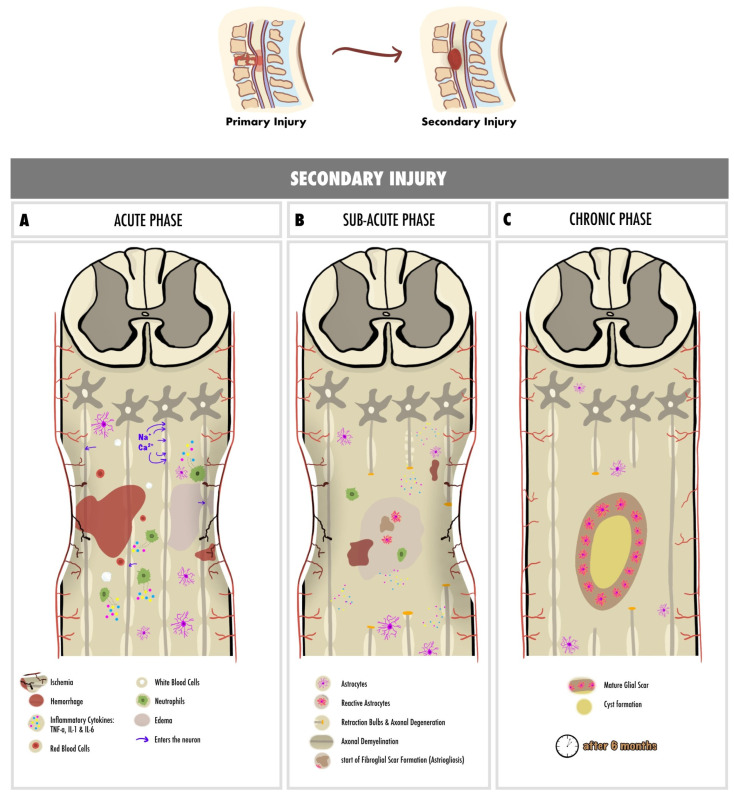
Spinal cord injury phases’ pathophysiology: (**A**) acute phase pathophysiology marked by ischemia, hemorrhage, red and white blood cell recruitment, and inflammatory cytokine secretion by neutrophils; (**B**) sub-acute phase marked by Wallerian degeneration and retraction bulbs formation, axonal demyelination, and the beginning of astrogliosis; (**C**) chronic phase marked by glial scar and cyst formation. TNF-α: tissue necrosis factor alpha, IL-1: interleukin-1, IL-6: interleukin-6.

Figure 1 represents a schematic representation of the pathophysiology of spinal cord injury, with a detailed summarization of each phase of secondary injury.

## 3. Adipose-Derived Stem Cells

Embryonic and adult stem cells are the two main types of stem cells. These cells have a characteristic ability to self-renew and differentiate along different cell lineages [37]. Mesenchymal stem cells are one type of adult stem cells that can be isolated from several different types of tissues, including the umbilical cord, endometrial polyps, brain, bone marrow, or adipose tissue, among others. Taking into account the large numbers of cells that can be harvested, as well as the feasibility and practicality of the process, these cells were targeted for their potential use in clinical applications [38].

Adipose tissue-derived stem cells (ADSCs) are isolated from either white or brown adipose tissue, and each cell has its unique characteristics. ADSCs from different anatomical locations also exhibit distinct characteristics [39]. ADSCs have the ability to differentiate into adipocytes, chondrocytes, myocytes, and osteoblasts, which made them a target for many clinical trials in different diseases, including diabetes mellitus and liver disease [40,41]. ADSCs also have the potential to differentiate into neurocytes, which makes them a possible therapeutic target for neurologic disorders [41].

Adipose tissues, though mesodermal in origin, can undergo differentiation to mesodermal or trans-mesodermal lineages, and they can give rise to cells of ectodermal origin [42]. Tissue regeneration by ADSCs is in part mediated by growth factors released by these tissues, namely basic fibroblast growth factor (bFGF), vascular endothelial growth factor (VEGF), insulin-like growth factor 1, hepatocyte growth factor (HGF), and transforming growth factor (TGF)-β1 [43]. Not only do target tissues express receptors for these growth factors, but ADSCs also express some receptors, and this allows for more effective regeneration and differentiation in response to a specific growth factor [44].

ADSCs were shown to have potential beneficial roles in the treatment of several neurological disorders or symptoms. They were shown to confer neurological improvement when used following an ischemic stroke and were demonstrated to aid in neuroinflammation modulation in rat models with sciatic nerve constriction [45,46]. They are also being studied for their ability to aid in the treatment of some neurodegenerative disorders where no treatments have yet been found, such as Alzheimer’s disease, Parkinson’s disease, Huntington’s disease, and amyotrophic lateral sclerosis [47].

ADSCs are able to differentiate into the neuronal lineages through a complex regulatory mechanism that includes a neurotrophic effect, regulation of the microenvironment, and regulation of the signaling pathways [48]. As for the neurotrophic effect, they are able to secrete many factors which accelerate neuronal differentiation. BDNF, GDNF, CNTF, and neurotrophin-4 are neuroprotective and neurotrophic factors secreted by ADSCs that enhance neural regeneration and reduce muscle atrophy [49,50]. Other secreted factors, HGF, TGF-β, and VEGF, aid in angiogenesis and inhibit apoptosis [51,52]. Additionally, ADSCs led to the activation and proliferation of a neuroprotective phenotype of microglia, which decelerates the development and progression of some neurodegenerative disorders, such as traumatic brain injury, by downregulating neuroinflammation [53,54].

With regards to the microenvironment, ADSCs inhibit the inflammatory response by secreting angiogenic factors, allowing for tissue recovery and regeneration [55,56]. They also impose an anti-apoptotic effect through releasing anti-inflammatory factors and cytokines, including TSG-6 and STC-1, allowing for a stable microenvironment for neuronal differentiation from ADSCs [57,58].

As for signaling pathways, ADSCs regulate the activation/ phosphorylation of the Wnt5a/JNK signaling pathway, therefore activating neuronal differentiation [59]. Similarly, the BDNF/TrkB signaling pathway promotes neuronal differentiation via secretion of BDNF, while the ROCK signaling pathway inhibits neuronal differentiation of ADSCs [60,61].

Concerning SCIs, several translational and clinical studies confirmed the role of ADSCs in managing SCI, but no definitive results have been reached. Thus, further investigations and trials are necessary to explore the efficacy and adequacy of using ADSCs as a potential treatment for currently untreatable SCIs.

## 4. Animal Models for ADSC in SCI

Animal models play a crucial role in advancing medical knowledge and mitigating human suffering, serving as a cornerstone for our fundamental understanding of disease pathophysiology and human anatomy [62]. These models are designed with the specific aim of closely mirroring human tissue and physiology, thereby contributing significantly to preclinical investigations and laying the groundwork for advancements in healthcare [63].

The choice of an appropriate animal model for translational research and cell-based therapy depends on various factors, such as availability, ease of handling, anatomical and functional similarity to humans, and the specificity of the study’s focus [64]. The recent literature highlighting the impact of mesenchymal stem cells (MSCs) suggests a specific preference of certain species in addressing specific diseases [64].

In the context of SCI models, rats emerge as the most commonly used species, followed by mice, rabbits, dogs, cats, and pigs [65]. Rodents, particularly rats, are currently in high demand for initial SCI experiments due to their suitability for monitoring physiological parameters and pathological events following SCIs. Notably, rat studies have yielded insights leading to potential therapeutic strategies for SCI patients. The induction of injury in rat models, whether through spinal contusion, weight drop, or epidural balloon compression, results in tissue degeneration at the injury epicenter, extending to adjacent segments.

Comparatively, inducing SCIs in dogs represents an intermediate step between rodent models and human clinical trials. Pre-clinical trials using chondrodystrophic dog models with spontaneous acute SCIs offer a unique opportunity to reaching effective treatments [64]. This approach provides valuable insights that bridge the translational gap, facilitating a more nuanced understanding of therapeutic strategies for SCI. Moreover, non-human primates, due to their genetic, biological, and physiological similarities to humans, hold significance in evaluating potential therapies for SCIs. However, ethical concerns and high operating and care costs may limit their use [66].

One significant limitation of the use of animal models for SCI research is the loss of bladder function, necessitating manual bladder release during the initial 7–10 days after injury. Although animal studies often underutilize bladder function assessment, simple procedures can be employed to evaluate dysfunction over time [67]. Understanding the various aspects of the pathology in animal models and correlating them with human patient pathology is crucial. While acknowledging the limitations, animal models remain an irreplaceable and crucial tool in SCI research.

It is essential to recognize that these models cannot fully replicate all the complexities of human SCIs, underscoring the importance of defining specific research questions and ensuring that the chosen model is fit-for-purpose. Careful selection and design of animal models is paramount for any translational drug development strategy [66].

Regarding the location of injury in spinal cord research, the thoracic region was found to be the most commonly studied, followed by cervical, lumbar, and sacral regions [65]. In terms of injury patterns, contusion emerged as the predominant type, involving a brief focal force applied to the exposed spinal cord to induce damage. Contusion devices are specifically designed to cause transient and acute injury [68,69]. Conversely, compression models employ sustained force or displacement over an extended period, with the use of clips, balloons, solid spacers, expanding polymers, remotes, weight drops, calibrated forceps, screws, and straps, simulating the compression seen in scenarios like fracture dislocations and burst fractures [69,70]. Distraction models introduce relative axial translation of the vertebrae, mimicking tension forces experienced during SCIs [69,71]. Dislocation models, such as those developed by Fiford et al. and Choo et al., replicate vertebral displacement mechanisms to simulate column and anterior dislocations seen in traumatic human SCIs [72,73]. Transection models involve partial or complete slicing of the spinal cord in the transverse plane, offering benefits for assessing axonal regeneration and functional recovery. However, these models are less suitable for investigating the intricate pathophysiology of SCI, as spinal cord transections are uncommon in clinical settings [68]. Chemical models utilize various chemicals to mimic specific aspects of the secondary injury cascade following traumatic SCI. These models have proven valuable for exploring molecular mechanisms and the effects of therapies on particular pathways associated with SCIs [68].

**Figure 2:**
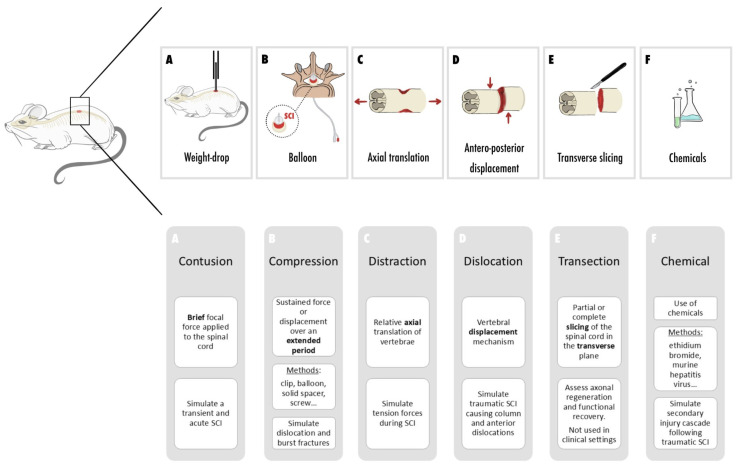
Injury patterns in SCI animal models (**A**) SCI induction by contusion (**B**) SCI induction by compression (**C**) SCI induction by distraction (**D**) SCI induction by dislocation (**E**) SCI induction by transection (**F**) SCI induction with chemical.

Figure 2 represents the different patterns of injury to the spinal cord.

## 5. Application Methods of ADSC in SCI

### 5.1. Fat Harvesting Sites

Many sites for harvesting adipose-derived stem cells have been proposed, and studies have aimed at identifying the most preferable sites. The abdomen and thighs are considered superior to other sites with regards to the number of cells harvested [74]. However, they can also be harvested from the infrapatellar Hoffa’s fat pad, flank, hip, or axilla [75,76]. Inguinal, epididymal, peri-renal, and back subcutaneous fats are also potential sites [77]. Surgery and direct excision, liposuction, or Coleman technique, a fat grafting technique used for fat transplantation and remodeling, are usually used for harvesting along with [78,79].

### 5.2. Isolation of Mesenchymal Stem Cells from Adipose Tissue

Initially, primary methods of isolation were developed by Rodbell and colleagues in the 1960s [80]. The procedure was then modified to a humanized version [81,82]. First, fragments of human tissue are minced by hand, but this procedure is simplified in liposuction, where the same result is obtained through the infusion of the subcutaneous tissue with a saline-containing anesthetic with or without epinephrine. They are given by a cannula, and both the liquid and the tissue are then removed under suction. The size of the minced fragments in this case depends on the size of the cannula [83]. Within the minced fragments, adherent stromal cells with adipocyte progenitor characteristics are then found in the liposuction aspirate and in the SVF (stromal vascular fraction). Centrifugation at a speed of 1200 g follows, allowing for optimal cell recovery [84]. Adipose tissue is then collected using needle biopsy or liposuction aspiration, and it must not be kept at room temperature for more than 24 h. Next, ADSCs are isolated from the adipose tissue. Initially, the tissue is washed thoroughly using phosphate-buffered saline (PBS) with 5% penicillin/streptomycin (P/S). Next, debris are removed, and the sample is placed on a sterile culture plate containing 0.075% collagenase Type I in PBS with 2% P/S. The tissue is then digested and ready to be minced using two scalpels and a pipette. The following step is incubation. The sample must be incubated at 37 °C and 5% CO_2_ for 30 min. Collagenase Type I activity is then neutralized by 5 mL of α-MEM and 20% heat-inactivated fetal bovine serum (FBS, Atlanta Biological, Atlanta, GA, USA). Pipetting follows for further digestion, and the sample is then transferred to a 50 mL tube, disallowing solid aggregates. The sample is centrifuged at 2000 rpm for 5 min, and the SVF is subsequently obtained. This is followed by vigorous shaking for disruption of the pellet and mixing of the cells. At this stage, stromal cells must be completely separated from the adipocytes. Centrifugation is repeated, and the collagenase solution above the pellet is then aspirated cautiously not to disturb the cells. At this point, the pellet must be resuspended in 1 mL lysis buffer, incubated on ice for 10 min, washed with 20 mL PBS with 2% P/S, and centrifuged at 2000 rpm for 5 min. Next, the supernatant is aspirated and the cell pellet is put in no more than 3 mL of stromal medium with 20% FBS, 1% L-glutamine, and 1% P/S, and the cell suspension is filtered on a 70 mm cell strainer. The strainer is then washed with stromal medium to obtain any residual cells before plating on a lysine-coated culture plate, and this plate must be incubated at 37 °C, 5% CO_2_. Finally, the cells are inoculated in a single well of a 12 or 24-well plate [76,85,86,87].

### 5.3. Culture and Expansion of ASCs

A total of 72 h after completing isolation, the entire medium is aspirated from the wells. FBS is increased to 25% if SVF does not expand well, but this might cause premature adipogenesis. Here, checking for peri-nuclear granularity, cytoplasmic vacuolations, and/or detachment of the cells from the plastic surface must be conducted to ensure there is no toxicity to the medium, microbial contamination, or death of the primary cells [85]. At this stage, PBS is warmed to wash the cells, and 1% antibiotic can be used as well. Unwanted tissue fragments or blood cells are then removed by pipetting the solution over the cell layer several times. Next, fresh stromal medium is added, and the cells are kept in a humid incubator at 37 °C with 5% CO_2_. After 80–90% confluence is attained by changing the medium every 2 days, the cells are either harvested or destined for adipocyte differentiation. ADSCs can be harvested by first adding and keeping 250–500 μL of sterile, warm PBS on the cells, which is then replaced by 500 μL of Trypsin/EDTA solution (0.5%). They are then incubated for 5 min till attachment is assured using microscopy. Once a minimum of 90% of the cells are detached, 500 μL of stromal medium are added for neutralization of the trypsin reaction. The following step requires moving the yield to a sterile 2 mL tube, and centrifuging at 1200 rpm for 5 min. After aspirating the supernatant and suspending the cells in approximately 250 μL of stromal medium, cell counting must be performed. A sample is diluted with trypan blue, and the cells are counted using a hemocytometer [85,88].

At this point, the cells can be cultured on culture plates. If the cells were expanded from a frozen vial, they must be thawed at 37 °C, and immediately then directly seeded on a plate with complete stromal medium, which must be changed the next day then every second day [85].

### 5.4. Differentiation

When ADSCs are destined for differentiation, the following steps are required; first, the prepared medium is aspirated and mixed with a small volume of warm PBS and 1% antibiotic. In this manner, the cells are washed, and PBS can be aspirated again for removal. ADSCs are pre-incubated with a pre-induction medium of 5 mM β-mercaptoethanol (β-ME) for 24 h [89]. The well is kept dry and the differentiation medium is added [85].

### 5.5. Neural Differentiation of ASCs

At high density, cellular spherical clumps are generated from cultured undifferentiated ADSCs. These can be isolated using 0.25% trypsin/2.21 mM EDTA. Free-floating neurospheres are also released and can be collected and cultured on an ultra-low cluster culture disk with neurobasal medium, where the spheroids must be kept at 10–20 per cm^2^ to avoid aggregation. Neural induction medium (NIM) of 2% dimethyl sulfoxide (DMSO), 200 µM butylated hydroxyanisole (BHA), and 40 ng/mL basic fibroblast growth factor (bFGF) are added to the sample which is replenished every 4 days [89]. An alternative NIM contains B27 (1:50), 20 ng/mL bFGF, and 20 ng/mL EGF, where the culture is left for 4 to 7 days. In this case, the neurospheres are put on Nunc culture plate with B27, and 70% of the medium is replaced every 3 to 4 days.

Some antigens expressed on differentiated neuronal cells, which can be tested to confirm the differentiation. They include nestin, NeuN, intermediate filament, MAP2, β-III tubulin, glutamate receptor subunits NR1 and NR2, oligodendrocyte marker, S-100, or glial fibrillary acidic protein (GFAP), and these markers can be detected by RT-PCR, immunohistochemistry, or Western blots [85].

### 5.6. Transplanting ADSC (Injection; Surgical Procedures; Intrathecal vs. Intravenous; Amounts)

Intrathecal ADSC administration is more efficiently used in SCI as compared to intravenous administration. This is explained by easy dispersion of the cells when given intravenously, with only a minimal amount reaching the spinal cord and crossing the blood–brain barrier [90].

According to one study, 3 μL of saline- or GFP-labeled ADSCs (1 × 10^6^ cells) was administered during the operation [91]. Other studies indicated similar amounts of the order 10^6^ [92].

According to Feron et al., the following procedure for ADSC transplantation into a patient with SCI was adopted [93].

#### 5.6.1. First Transplantation

Patients undergo surgery for spinal cord fixing and decompression, and the transplant is then administered. General anesthesia is given, and lidocaine is used at the incision site. A pre-operative X-ray is performed to locate the lesion site, and therefore the transplant site. During the operation, the following steps are followed: first, a midline incision is made at the vertebral column rostral and caudal to the injury site. Second, rostral and caudal multiple-level laminectomies of the dura are performed. This is followed by a midline durotomy. Next, dorsal adhesiolysis assisted by microscopy is conducted through blunt dissections at the injury site. The last step includes the delivery of 2 mL of the SVF solution to the subarachnoid space above, central to, and below the injury site. Before closing, the rest of the SVF solution is administered into the subarachnoid space [93].

#### 5.6.2. Second and Third ADSC Transplantation

Using the same technique, a second transplant is carried out on day 30, followed by a third on day 45.

#### 5.6.3. Fourth ADSCs Transplantation

The last injection is administered to the spine on day 75.

The administration of ADSCs targets several mechanisms promoting the SCI’s recovery.

**Figure 3:**
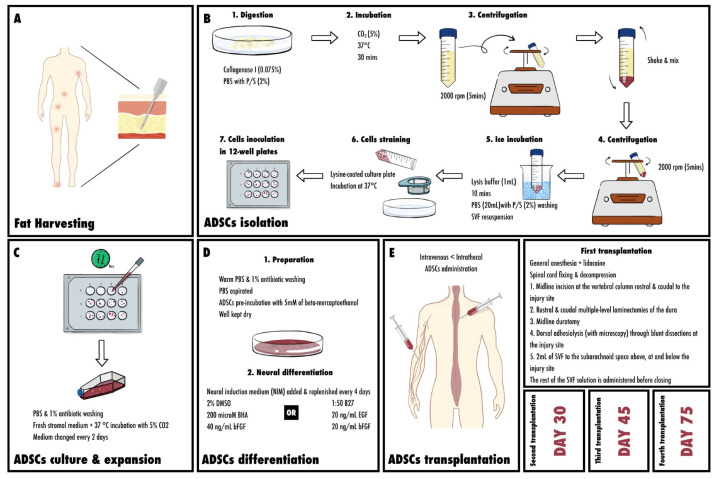
ADSC collection and transplantation in SCI: (**A**) fat harvesting; (**B**) ADSC isolation; (**C**) ADSC culture and expansion; (**D**) ADSC differentiation; (**E**) ADSC transplantations. PBS: phosphate-buffered saline, P/S: penicillin/streptomycin, CO_2_: carbon dioxide, rpm: round per minute, SVF: stromal vascular fraction, DMSO: dimethyl sulfoxide, BHA: butylated hydroxyanisole, bFGF: basic fibroblast growth factor, EGF: epidermal growth factor.

Figure 3 represents the process of ADSCs in spinal cord injury.

## 6. Inflammation, Nociception and Neuroprotection

Considering the implication of ADSCs in SCIs recovery, their inflammation-related actions should be highlighted. CINC-1, a chemokine that is increased after adipose cell transplantation, eliminates injured cells, cell debris, and ECM proteins by activating and infiltrating neutrophils, which prepares for tissue regeneration [94]. Previous studies have revealed that ADSCs exert several anti-inflammatory effects by downregulating pro-inflammatory cytokine expression, like IL-1β and TNF-α, and by upregulating the expression of other anti-inflammatory agents like TSG-6 [95,96]. In the same context, adipose stem cell therapy has shown a significant lowering in the expression of several inflammatory and oxidative stress proteins, such as NOX-1, NOX-2, TLR-4, MyD88, substance-p, Mal, TRAF6, IKK-α, IKK-β, and NF-κB [95]. This study has also demonstrated the ADSC-induced reduction of post-SCI inflammation by the downregulation of CD68 and GFAP cellular inflammatory markers expression. Moreover, ADSC therapy in SCIs decreased IL-6 secretion, a well-known inflammatory cytokine, as well as AQP4 secretion, a neuroinflammation indicator whose production is related to that of IL-6 [58,96,97,98].

The suppression of IL-6 and AQP4 production, in addition to the ADSC-associated increase in GDNF and GABA receptors (1 and 2), both involved in pain control, explained the higher paw withdrawal threshold after mechanical, cold, and heat stimulations in rats that underwent ADSCs transplantations, indicating that this intervention enhanced pain tolerance [99,100,101,102,103,104]. Yin et al. found that the expression of the cell-stress signals PI3K, (p)-AKT, and p-mTOR, as well as that of Nav1.3, Nav1.8, and Nav1.9 voltage-gated sodium channel markers, was amplified, while the expression of some mitogen-activated protein kinase (MAPK) family biomarkers was diminished, confirming once again the improved SCI pain endurance after ADSCs administration [95].

Besides inflammation and nociception reduction, fatty stem cell injection in SCI cases presented a noteworthy neuroprotective effect. One of the most interesting findings was the association with SCI inflammation, where cellular therapy restricted the macrophage diffusion along the spinal cord and limited it to the lesion borders, therefore promoting tissue sparing by protecting the healthy parenchyma. This process was ascribed to the secretion of laminin proteins [105,106]. Moreover, lesion cavities were smaller in ADSC-treated groups and less scar formation was detected in them because of lower GFAP and higher CNTF levels, which assures tissue regeneration without forming any scars [105,107,108]. In one study, rAT-MSCs decreased the number of apoptotic cells at the lesion site when injected in both the acute and sub-acute phase of the SCI. In this framework, adipose stem cells have been reported to enhance ERK1/2 and Akt phosphorylation, which might play a role in their antiapoptotic effect [94]. Likewise, CINC-1 is a chemokine that can protect neurons from apoptosis and its expression was actually increased 3 h after ASC injection [94,109]. Motor and sensory neuron survival was potentially enhanced by the increase in GDNF, which regulates the levels of XIAP and NAIP (Cohen’s d = 5.335) [98,100].

## 7. Neurogenesis, Neuronal Regeneration and Axonal Regrowth

Post-SCI injury, ADSCs play an efficient role in regenerating the neural tissue. In fact, they are capable of activating axonal regrowth, particularly that of serotonergic descending axons [105]. Given that the extracellular matrix has the ability in axonal regeneration and in minimizing inflammation along the spinal cord, hADSCs (human adipose-derived stem cells) optimize neural regeneration [106,110,111,112]. This is executed by increasing the neural precursors’ abundance via the secretion of laminin proteins, consequently promoting cellularity at the SCI site in addition to distributing new cell clusters above the spinal canal that are subsequently recruited to the lesion epicenter, therefore camouflaging the cellular loss [105]. It is noteworthy to mention that ADSC transplantation manifests an increase in β3-tubulin expression, which is an early indicator of neuronal differentiation [107,108,113].

Moreover, these murine and human adipose stem cells have the capacity to undergo morphologic and phenotypic changes after neuronal induction, resembling the neuronal perikaryal appearance: spherical and refractile cell bodies with prolonged cytoplasmic extensions. However, these changes do not persist. Eventually, by 14 days, all the neuronal-looking cells are dead [114]. Ohta et al. demonstrated the same process in their study. The smaller cavities at the site of the SCI after an intravenous infusion of ADSCs led them to further investigate their potential differentiation into neural progenitors, particularly since these fatty cells can express Tuj1, Olig2, and many other neural markers [94,105,115]. The ability of these stem cells to differentiate into neuronal or glial cells was proven in vitro, but when tested in vivo, they lived shortly then died [94,114,116]. This means that, till now, fatty stem cell transplantation alone has failed to fully differentiate into mature long-living neural cells, and further studies should be held in order to prolong the survival of those differentiated. Acknowledging these limitations, the use of a cell-adaptable neurogenic (CaNeu) hydrogel might be beneficent since it enhances the neural differentiation of encapsulated ADSCs by increasing their YAP expression, which is proven to affect stem cells viability, expansion, and differentiation [117,118]. Similarly, the co-administration of ADSCs with arginine, alanine, aspartic acid, and glycine in a hydrogel has also shown expanded ADSCs survival at the site of injury [119].

Concerning the role of BDNF in neurogenesis, study results diverge on this matter. While some studies have found that BDNF has effectively stimulated axonal regrowth and neuron regeneration by adipose stem cell neurodifferentiation, Sarveazad et al. (2019) reported that the hADSCs’ ability to regenerate neurons is probably caused by other BDNF-independent mechanisms, such as those stimulated by the increase in GDNF [98,107,120].

## 8. Angiogenesis and Vascularization

One of the main mechanisms through which ADSCs transplantation promotes SCI recovery is angiogenesis. Two weeks after transplanting them, they efficiently increase the number of vascular endothelial cells at the spinal cord’s midsagittal area [121]. Adipose stem cell treatment in mice with ischemic hindlimbs effectively increases the release of VEGF (Cohen’s d = 6.164), which contributes to improving perfusion by enhancing endothelial cells’ survival and proliferation, especially since VEGF is instrumental in vascular networks formation [52,122]. Furthermore, neocapillaries were detected on sponges treated with CINC-1, a chemokine that was increased after ASC injection, confirming once again its role in vascularization [94,123].

Additionally, a previous review explained the remarkable role of pericytes in blood vessel regeneration, maturation, and maintenance [124]. Indeed, several studies examining the reparative abilities of hADSCs in rats with spinal cord compression proved their angiogenic effect by endogenous pericytes’ recruitment into the site of injury. New mature blood vessels were detected with two separated basement membranes along the spinal cord axis, between which a perivascular space was formed and accumulated cells expressing pericytes markers, such as αSMA, vimentin, PDGF-Rβ, nestin, and NG2 [105,125].

## 9. Outcomes (Studied Factors with the Outcomes Reached)

### 9.1. Locomotion and Functional Recovery

One of the goals of fatty stem cells injection in rats following an SCI is to enhance their locomotor ability. A systematic review and meta-analysis showed that ADSC transplantation improved the locomotor ability following SCI no matter how many cells were injected and independently from the injury’s location, severity, model, and treatment phase [126].

In order to assess hindlimb coordination and functionality in SCI rats, a Basso, Beattie, and Bresnahan (BBB) score was often used, comparing the rats’ open field locomotion with a scale ranging from 0 (complete paralysis) to 21 (normal motricity) [127]. Aras et al. recorded the motor ability before and on several dates after the injury, with and without ADSC treatment. Although spontaneous recovery was perceived in untreated rats too, adipocyte transplantation accelerated the process, scored a higher BBB, and presented less motor damage [107]. Likewise, other studies have detected the same findings and have found normal hindlimb motility supporting the rat’s body weight, as opposed to the untreated SCI rats whose limbs failed in lifting their weights and consequently had abnormal crawling [94,105,125]. Interestingly, although the BBB score post-treatment is usually still below the normal value, a study experimenting on moderate compressive SCI rats reported a score almost identical to that seen in normal healthy rats 4 weeks after ADSC injection [105,128].

Notably, experiments on canine models exhibited similar efficacy in response to this therapeutical approach, not only rodents. Using the Olby score, a 15-point scoring system, SCI dogs’ gaits and mobility were assessed and have manifested a significant improvement after ASC injection, having moveable pelvic limb joints, with a score of 4.6 nine weeks after the start of treatment [129]. Conversely, dogs receiving no treatment or saline buffers had a score below 1, indicating no pelvic limb movement [130]. Despite this, ASC transplantation was not as impactful as that of umbilical cord blood-derived stem cells which has a reported Olby score of 7.4 [131].

Acknowledging that adipose stem cell therapy alone has shown several limitations and has not reached the fully expected motor recovery yet, researchers endeavored to solve this issue in their studies. Eventually, motor-evoked potential was further enhanced with the incorporation of cell-adaptable dynamic (CaNeu) hydrogel as a delivery vehicle, with a BBB score increasing from 4.5 ± 3.2 in the ADSCs group to 9.2 ± 2.0 in the CaNeu/ADSCs group (Cohen’s d = 1.76) [117]. Moreover, the addition of hyperbaric oxygen (HBO) to ADSCs could be promising, considering the remarkably improved locomotion seen with combined therapy (ADMSCs-HBO). Hence, utilizing HBO with the adipocytes has been suggested in the treatment of traumatic SCIs, especially in cases that are irresponsive to conventional management [95]. Similarly, in a study aiming to improve the transplantation’s outcomes, Ohta et al. have observed a better functional recovery when using a graft of three-dimensional cell mass from hASC-derived endothelial lineage cells (3DCM-ASCs), compared with the injection of adipose stem cells alone [132]. Low-level laser co-administration has also shown a significant enhancement in motor function restoration (*p* = 0.0001) [98]. Furthermore, improvements in mobility and muscular coordination were demonstrated in rats combining ADSC grafts with functional rehabilitation in treadmills [121].

### 9.2. Neural Relay Restoration, Sensory Rehabilitation and Pain Reduction

On account of the nerve damage that usually accompanies a spinal cord injury, SCI treatments should also aim to restore the normal somatosensorial pathways and fix any sensorial impairment, pain included. This was evaluated by the assessment of somatosensory potentials, which were successfully evoked in SCI dogs receiving ASCs, while no response at all was registered in untreated dogs (Cohen’s d = 0.717) [130]. Similarly, electrophysiological analysis in SCI rats showed an improved mean evoked potential (MEP) after a cell-adaptable dynamic (CaNeu) hydrogel-mediated ADSC delivery (Cohen’s d = 1.264) [117].

To examine the graft’s effect on the SCI rats’ sensitivity to mechanical and thermal stimuli, several tests have been established. Yousefifard et al. used the Von-Frey, acetone, Randall-Selitto, and plantar test to measure mechanical allodynia, cold allodynia, mechanical hyperalgesia, and heat hyperalgesia, respectively. As a result, the paw withdrawal threshold for all factors was significantly increased post-adipocyte transplantation (*p* < 0.0001) when compared to the injured rats that manifested a remarkably decreased pain threshold. Notably, the paw withdrawal threshold could not be returned back to its normal level in healthy rats using the graft [128]. They also observed similar findings in an older study using laser co-administration. However, this study differed in that the mechanical hyperalgesia was significantly improved with laser + hADSC combined therapy (*p* < 0.0001), but not in the groups receiving hADSCs exclusively (*p* > 0.99). These results were different from those in their most recent study [98].

### 9.3. Neurogenic Bladder Recovery

While neurogenic bladder is a frequently encountered spinal cord injury complication, most studies have neglected this issue and focused more on functional motor and sensory recovery. Chen et al. dedicated their study to investigate the ability of ADSC sheets transplantation to restore normal bladder function. The voiding function was evaluated based on the measurement of some cytometric parameters (bladder capacity, voiding efficiency, peak bladder pressure, residual volume…), and was significantly enhanced following treatment. The different layers of the bladder’s urothelium are normally characterized by the expression of specific markers [113]. Basal cells positioned along the basement membrane can be identified by krt5 and p63 coupled, with the absence of Upk and krt20. Intermediate cells are positioned above the basal cells and express p63 and Upk, but do not express krt20 and krt5. Umbrella cells express uroplakins (Upk) and krt20, but lack expression of p63 and krt5 [133]. These markers were altered in the case of SCI rats, mainly losing krt20+ umbrella cells. Cellular therapy has gradually reversed this alteration. Lastly, the transplantation of adipocyte sheets has successfully restored the normal bladder musculature [113].

## 10. Clinical Application

In December 2023, a search was conducted on clinicaltrials.gov, using “Spinal cord injury” as the Condition/disease and “Adipose-derived stem cell” as the Intervention/Treatment [134]. The results, summarized in Table 1, revealed a total of 11 clinical trials. Among these, there were four phase I trials, four phase I/II trials, one phase II trial, and two trials that did not mention their phase. The combined enrollment of participants across these trials was 212, with individual trial enrollments ranging from 8 to 48 participants. The duration of the studies ranged between 1 and 7 years. Notably, two trials are currently ongoing, with one still in the recruitment phase and the other suspended.

The Republic of Korea was a pioneer in conducting clinical trials on AD-MSC for SCI, with three trials started in 2009, 2012, and 2013. The United States leads in terms of the total number of clinical trials, accounting for four trials, although data for two of these trials are no longer available.

Table 2 provides a comprehensive overview of clinical trials utilizing AD-MSC injections as a therapeutic approach for SCI. The table outlines key information for each trial sourced from clinicaltrials.gov, including study design, primary objectives, inclusion criteria, and intervention details. Out of the 11 clinical trials identified, only three have corresponding published articles in the scientific literature [135,136,137].

Ra, J.C., et al. comprehensively explored the safety and efficacy of AD-MSC for SCI treatment, starting with a preclinical in vitro study on animal models, followed by a phase I human clinical trial [135]. The investigation confirmed the genetic stability of AD-MSC through 12 subcultures, supported by normal karyotypes and the absence of genome abnormalities. In physiological saline, MSCs remained viable for at least 72 h, showcasing their potential for clinical use. Toxicity assessments in mice demonstrated the safety of AD-MSC, with no adverse events at doses exceeding 2.5 × 10^8^ cells. In vivo distribution in an SCI rat model revealed significant presence in the spleen, thymus, and unexpectedly, the spinal cord [138]. Tumorigenicity tests showed no tumor formation over 26 weeks, aligning with prior studies [139]. Regarding the human clinical trial, findings confirmed the safety of intravenous administration of autologous AD-MSC, with observed adverse events, such as headache, typical for SCI patients which improved spontaneously or with medication [140]. The efficacy assessments in SCI patients revealed noteworthy neurological advancements, including a remarkable transition from ASIA grade A to C in one patient and favorable enhancements in motor scores [27]. Notably, the improvement in pin-prick scores exceeding that of light-touch scores suggests a positive outlook for functional ambulation, underscoring the promising potential of AD-MSC as a viable treatment for SCI.

Hur, J.W., et al. conducted an open-label clinical trial with factorial assignment to assess the effect of intrathecal AD-MSC injections in SCI patients [136]. Among the 14 patients in the study, 5 individuals exhibited motor improvement based on the ASIA motor score. Notable cases include a 66-year-old male with T12-level injury, experiencing a score increase from 50 to 56 six months after AD-MSC injection. Moreover, a 58-year-old female with a C5–6 injury progressed from ASIA grade D to an ASIA motor score of 77 at the 2-month follow-up. Additionally, voluntary anal contraction improvements were observed in two patients, while sensory improvements were noted in ten patients, with only one experiencing sensory regression. MRI and electrophysiological assessments demonstrated no significant changes in spinal damage, indicating the absence of tumorous or calcification signs. No serious adverse events related to intrathecal ADMSC administration were observed during the 8-month follow-up, reinforcing the overall safety and potential efficacy of the treatment.

Bydon, M., et al. presented a report of the first treated patient in the CELLTOP clinical trial, aiming to determine the safety of AD-MSC injection in the spinal fluid [137]. The patient was a 53-year-old male who suffered a surfing accident resulting in a C3–4 grade A SCI. The study results demonstrated the safety and positive outcomes of the procedure, with the patient tolerating the injection well and experiencing only a mild to moderate headache that resolved with acetaminophen. No serious adverse events or safety issues were reported throughout the 18-month follow-up period. The ASIA motor score exhibited progressive improvement, particularly in the upper extremities, with the total score increasing from 35 to 44 at 18 months. Notable improvements were observed in both upper and lower-extremity motor scores bilaterally. The ASIA sensory score consistently improved, with the pinprick and light-touch scores showing significant enhancements on both sides. Dermatomal regions, particularly C5 in the upper extremity and L4, L5, and S1–4 in the lower extremity, displayed substantial improvement. Additionally, the Global Health Score and physical and mental health measures reflected substantial improvements in the patient’s overall quality of life. Physical therapy and occupational therapy assessments demonstrated considerable progress in walking speed (from 0.17 m/s to 0.43 m/s at 15 months), ambulation distance (from 635 ft for 12.8 min at a pace of 0.25 m/s at baseline to covering 2200 ft for 34 min at a pace of 0.33 m/s at 15 months), and range of motion for flexion and abduction of the shoulder joint. These findings collectively support the safety, motor and sensory improvements, and enhanced quality of life associated with the intervention.

## 11. Challenges and Future Perspectives

ADSC therapy holds a big promise for shaping treatment strategies for SCI. However, numerous challenges still impede its implementation in clinical practice. Despite being comparable to other types of stem cells, these types of stem cells possess some unique challenges and advantages.

A key challenge in ADSC therapy stands at the level of isolation and culture methods, affecting the safety and purity of the cells. Additionally, identifying the best source and dose contributes to enhancing the results of therapy, besides finding the optimal timing. Optimizing the differentiation and integration of stem cells into the host presents another promising area for future investigation, where gene-editing and tissue-engineering technologies can be of great benefit. Collaborative efforts between neuroscientists and bioengineers are needed to overcome the above-mentioned challenges.

Immunogenicity is another important point with a crucial role in the clinical use of ADSCs, where different immunomodulatory profiles are presented in the long-term [141]. Following manipulation and expansion of ADSCs, genetic stability, mainly affected by spontaneous transformation or accumulation of genetic alterations, remains a major concern [142,143,144]. Another point to address is the possibility of malignant events, which despite being extremely rare, have been reported in some cases [145].

Another challenge in transplanting ADSCs exists at the level of glial scar formation and neuronal loss, necessitating a better understanding of the SCI microenvironment. The combination of therapies, in addition to targeted drug delivery and tissue-engineering techniques, may aid in overcoming such adverse effects of ADSC therapy [146,147,148].

Overall, the huge progress reached in treating SCIs using ADSCs is still limited by a number of challenges, requiring additional interventions and more research to overcome therapeutic obstacles and improve efficiency. The combination of ADSCs with other treatments, such as electrical stimulation, offers a promising avenue for future research. Furthermore, research targeting genetic engineering technology, nanobiotechnology, and neuroprotective agents may help clinicians achieve better outcomes.

## 12. Conclusions

Despite maximal efforts, minimal progress has been made in the treatment of SCIs and their associated symptoms, rendering this disease a massive burden with reduced quality of life. Recently, the use of stem cells is being widely studied with the aim of reaching new and effective therapeutic strategies. Considering their benefit in other diseases, ADSCs may serve as a potential and valuable option for treating SCIs. However, the majority of research is still at the preclinical level, with minimal clinical applications. The advancements made using animal models, in addition to improvements in the techniques used for preparing and transplanting stem cells, aided in initiating more research with higher efficacy.

The significant role of ADSCs in neuroprotection and reduction in inflammation and nociception, in addition to their ability to trigger neurogenesis and enhance angiogenesis and vascularization, has encouraged researchers to move to clinical studies, especially with the positive results reached in terms of motor and sensory outcomes. Despite this, clinical research remains limited, highlighting the need for additional studies focused on human models. This review helps encourage researchers to further investigate the potential benefits of ADSCs in spinal cord injury and help establish a deeper understanding of the mechanism of action of ADSCs in SCI.

## Figures and Tables

**Table 1:** Characteristics of clinical trials on ADSCs in SCI found on clinicaltrials.gov.

NCT Number	Study Title	Study Status	Results	Sex	Enrollment	Phase	Start Date	Duration	Location
NCT02981576	Safety and Effectiveness of BM-MSC vs. AT-MSC in the Treatment of SCI Patients.	Completed	No	All	14	I/II	November 2016	3 years	Jordan
NCT02917291	Safety and Preliminary Efficacy of FAB117-HC in Patients With Acute Traumatic Spinal Cord Injury.	Unknown	No	All	48	I/II	December 2016	7 years	Spain
NCT02034669	Transplantation of Autologous Adipose Derived Stem Cells (ADSCs) in Spinal Cord Injury Treatment.	Unknown	No	All	48	I/II	February 2013	2 years	Vietnam
NCT01769872	Safety and Effect of Adipose Tissue Derived Mesenchymal Stem Cell Implantation in Patients With Spinal Cord Injury.	Completed	No	All	15	I/II	January 2013	3 years	Republic of Korea
NCT01624779	Intrathecal Transplantation Of Autologous Adipose Tissue Derived MSC in the Patients With Spinal Cord Injury.	Completed	Yes	All	15	I	April 2012	2 years	Republic of Korea
NCT01274975	Autologous Adipose Derived MSCs Transplantation in Patient With Spinal Cord Injury.	Completed	Yes	Male	8	I	July 2009	1 year	Republic of Korea
NCT05018793	Safety of Cultured Autologous Adult Adipose Derived Mesenchymal Stem Cell Intrathecal Injection for SCI.	Suspended	No	All	15	I	December 2012	4 years	Greece
NCT04520373	Autologous Adipose Derived Mesenchymal Stem Cells for Spinal Cord Injury Patients.	Recruiting	No	All	40	II	June 2020	4 years	United States
NCT04064957	Individual Patient Expanded Access IND of Hope Biosciences Autologous Adipose-derived Mesenchymal Stem Cells for Spinal Cord Injury.	No longer available	No	All	N/A	N/A	N/A	N/A	United States
NCT03925649	Individual Patient Expanded Access IND of Hope Biosciences Autologous Adipose-derived Mesenchymal Stem Cells for Treatment of SCI.	No longer available	No	Male	N/A	N/A	N/A	N/A	United States
NCT03308565	Adipose Stem Cells for Traumatic Spinal Cord Injury.	Completed	Yes	All	10	I	December 2017	4 years	United States

**Table 2:** Summary of clinical trials on ADSCs in SCI.

Title	Study Design	Primary Objectives	Inclusion Criteria	Intervention
Autologous Adipose Derived MSCs Transplantation in Patient with Spinal Cord Injury	Randomized Open Label Single group Assignment	Assess the safety of intravenous autologous AD-MSC transplant in SCI patients.	Males between 19 and 60 years. AIS grade A, B or C. Duration of injury: >2 months	Group 1: IV injection of AD-MSC (400 million cells) (Astrostem^®^)
Intrathecal Transplantation of Autologous Adipose Tissue Derived MSC in the Patients With Spinal Cord Injury	Open Label Factorial Assignment	Assess the effect of intrathecal transplantation of autologous AD-MSC in the patients with SCI.	Male or female aging between 19 and 70 years. No chance of improving neurological function despite performed the optimal treatment after SCI. No change in neurological function for 4-week intervals by at least 2 clinical medical specialists	Group 1: Intrathecal injections of AD-MSC (90 million cells) at day 1, after 1 month and after 2 months.
Safety and Effect of Adipose Tissue Derived Mesenchymal Stem Cell Implantation in Patients With Spinal Cord Injury	Open Label Single group assignment	Investigate the efficacy and safety of autologous transplantation of AD-MSC in patient with SCI	Male or female aging between 19 and 70 years. AIS grade A, B or C. Duration of injury > 3 months.	Group 1: IV and Intrathecal injections of AS-MSC.
Transplantation of Autologous Adipose Derived Stem Cells (ADSCs) in Spinal Cord Injury Treatment	Randomized Open Label Parallel Assignment	Assess the safety and effect of AD-MSC transplantation in acute SCI patients.	Male or female aging between 19 and 60 years. AIS grade A. Patients with complete spinal cord < 2 weeks in acute category.	Group 1: AD-MSC transplantation 4 Interventions: laminectomy, intradural space at damage site, intrathecal at lumbar puncture, and IV. Group 2: only laminectomy.
Safety and Preliminary Efficacy of FAB117-HC in Patients with Acute Traumatic Spinal Cord Injury	Randomized Double Masked Parallel Assignment	Evaluate the safety and tolerability of FAB117-HC (a medicinal product containing human AD-MSC expanded and pulsed with H_2_O_2_, HC016 cells) administered at a single-time point to patients with acute thoracic SCI.	Male or female between 16 and 70 years. AIS grade A and B. Single traumatic spinal cord injury as defined by MRI. Either a level of injury between D1–D12 both inclusive. Injury occurred between 72 and 120 h before undergoing DSS and treatment. Clinically and hemodynamically stable, under medical criteria, enough to undergo DSS.	Phase 1: (only grade A) Intramedullary injection of Drug: FAB117-HC (3 patients with 20 million cells and 5 patients with 40 million cells) Phase 2: (grade A or B) Group 1: Intramedullary injection of FAB117-HC (20 or 40 million cells) Group 2: No treatment
Safety and Effectiveness of BM-MSC vs. AT-MSC in the Treatment of SCI Patients.	Randomized Open Label Parallel Assignment	Assess and compare the safety and effectiveness of autologous BM-MSC vs. autologous AD-MSC in SCI patients.	Male or Female between 18 and 70 years. Complete SCI grade AIS A or B, or incomplete C. At least 2 weeks since time of injury.	Group 1: Intrathecal injection of AD-MSC 3 times. Group 2: Intrathecal injection of BM-MSC 3 times.
Adipose Stem Cells for Traumatic Spinal Cord Injury	Open Label Single group assignment	Determine if AD-MSC can be safely administered into the cerebrospinal fluid of patients with SCI.	Male or female older than 18 years. AIS grade A or B. SCI must be traumatic, blunt/non-penetrating in nature and not degenerative. SCI must be within two weeks and up to 1 year after the event	Group 1: Intrathecal injection of AD-MSC (100 million cells).
Autologous Adipose Derived Mesenchymal Stem Cells for Spinal Cord Injury Patients	Randomized Open Label Crossover Assignment	Investigate the safety and potential therapeutic effects of autologous, culture-expanded, AD-MSC intrathecal injections in the treatment of SCI.	Male or female older than 18 years. AIS grade A or B. SCI must be traumatic, blunt/non-penetrating in nature and not degenerative.	Group 1: Intrathecal injection of AD-MSC. Group 2: 6 months attending physical and occupational therapy. Then, intrathecal injection of AD-MSC.
Safety of Cultured Autologous Adult Adipose Derived Mesenchymal Stem Cell Intrathecal Injection for SCI	Open Label Single group assignment	Study the safety and efficacy of intrathecal injection of cultured autologous AD-MSC for the treatment of SCI.	Male or female. Presence of a diagnosis of SCI.	Group 1: Intrathecal injection of AD-MSC (100 million cells).

AD-MSC: Adipose-Derived Mesenchymal Stem Cells, BM-MSC: Bone Marrow Mesenchymal Stem Cells, SCI: Spinal Cord Injury, IV: Intravenous, AIS: American Spinal Injury Association (ASIA) Impairment Scale, DSS: Dynamic Stabilization System.
